# Mode localization in chiral periodic approximants of Fibonacci magnonic superlattices

**DOI:** 10.1038/s41598-026-44837-2

**Published:** 2026-03-27

**Authors:** J. Flores-Farías, P. Contreras-Gallardo, F. Brevis, N. Vidal-Silva, P. Landeros, R. Arias, R. A. Gallardo

**Affiliations:** 1 Departamento de Física, Facultad de Ciencias Físicas y Matemáticas, Universidad de Chile, Santiago, Chile; 2 Departamento de Física, Universidad Técnica Federico Santa María, Avenida España 1680, Valparaíso, Chile; 3 Departamento de Ciencias Físicas, Universidad de La Frontera, Casilla 54-D, Temuco, Chile; 4 Center for the Development of Nanoscience and Nanotechnology (CEDENNA), 9170022 Santiago, Chile

**Keywords:** Spin waves, Flat bands, Magnonic crystals, Dzyaloshinskii–Moriya interaction, Materials science, Physics

## Abstract

Deterministic magnonic systems based on Fibonacci superlattices provide unique opportunities for manipulating spin-wave dynamics beyond the capabilities of simple periodic metamaterials. In this work, one-dimensional periodic approximants of Fibonacci superlattices are studied in both chiral and nonchiral nanomagnets, where a deterministic aperiodic ordering derived from a Fibonacci sequence is implemented through spatial modulations of the saturation magnetization, perpendicular anisotropy, and the interfacial Dzyaloshinskii–Moriya interaction (DMI). A combination of plane-wave calculations and micromagnetic simulations consistently predicts the emergence of dispersionless flat bands at low frequencies, along with their associated spatial localization patterns. The results show that aperiodic modulation encodes modal selectivity, enabling spin-wave modes to localize in regions defined by material contrasts in anisotropy, DMI, and saturation magnetization. Furthermore, it is shown that the frequency window in which the flat bands are excited is determined by the dispersion of the corresponding continuous films and can be tuned by an external magnetic field. This tunability provides a direct means of externally controlling and enhancing the flat-band regime, thereby broadening the operational bandwidth of dispersionless modes. These findings establish periodic approximants of Fibonacci superlattices as promising platforms for reconfigurable, compact magnonic devices, such as filters, multiplexers, and logic elements, in which deterministic spatial-mode control and external-field tunability are essential.

## Introduction

Spin waves (SWs), the fundamental excitations of the magnetization field in magnetic media, have become central to the conceptual and technological foundations of magnonics and spintronics. Their ability to carry and process information without relying on charge transport makes them a suitable candidate for next-generation devices aimed at minimizing energy dissipation while maximizing operational speed and scalability^1–5^. A key challenge in magnonics is achieving precise spectral and spatial control over spin waves. Periodic magnetic structures, or magnonic crystals (MCs), have enabled such control through Bragg scattering and bandgap engineering^6–10^. By modulating magnetic parameters such as saturation magnetization, anisotropy, or geometry, these systems exhibit forbidden frequency bands that have allowed the envisioning of magnonic filters, resonators, and waveguides^11–24^. A similar phenomenology can be observed in nanomagnets hosting periodic magnetic textures, where the crystal-like periodic potential arises from the dipolar field originated by the nonuniform magnetization^25–27^.

Magnonic superlattices, created by the introduction of defects within a magnonic crystal, enrich the SW modal spectrum^28–30^. These engineered inhomogeneities give rise to localized defect-mode states that emerge within the spectrum’s band gaps, analogous to hyperfine structure in atoms, thereby enabling robust wave confinement. On the other hand, recent theoretical and experimental studies have shown that the inclusion of chiral couplings, particularly the dipolar and the interfacial Dzyaloshinskii–Moriya interaction (DMI), can further enrich the spin-wave spectrum by inducing nonreciprocity^31–35^, unidirectionality^36–39^, and enabling the formation of indirect gaps and flat bands^40–47^. Such flat bands appear in ferromagnets in contact with a periodic heavy-metal layer that induces interfacial DMI. The flat bands are linked to strong SW confinement in regions where DMI is present^34,45^. Such localized modes could facilitate novel functionalities including signal filtering, directional control of wave propagation, and logic operations, which are essential for the development of reconfigurable and miniaturized magnonic devices^48–59^.

While periodic crystals and superlattices offer powerful tools for spectral engineering, quasiperiodic systems offer greater structural complexity and functionality. One-dimensional (1D) Fibonacci magnonic quasicrystals exemplify this approach, as they exhibit deterministic aperiodicity and long-range order in the absence of translational symmetry. Their inflation-based construction leads to scale-invariant spectral features, including self-similar minibands and localized modes^60–65^. Unlike disorder-induced localization, which is random and difficult to control, localization in quasiperiodic systems is reproducible and structurally encoded^66–70^. The spatial inhomogeneity inherent in a Fibonacci sequence enables localized excitations to emerge across different regions of the structure, depending on the modes’ frequency and symmetry. In this sense, the geometry of the system could serve as a passive computing element that encodes information in its structure. This concept, sometimes referred to as structural computing^71^, highlights the intrinsic ability of quasiperiodic systems to encode frequency-selective behavior deterministically. In this context, recent wave-vector-resolved Brillouin light scattering (BLS) experiments on chiral magnonic quasicrystals based on Ir/Fe/Pt(Ir) and Au/Fe/Pt(Au) multilayers have provided experimental evidence of these features, reporting tunable nearly flat bands, pronounced nonreciprocity arising from tailored interfacial Dzyaloshinskii–Moriya interaction, and fractal dispersion characteristics^72^. Beyond the Fibonacci sequence, other quasiperiodic patterns—such as two-dimensional Penrose tilings—have been demonstrated experimentally to support magnonic band structures and directional mode emission^73–80^. Theoretical studies of various aperiodic sequences, such as the Thue–Morse sequence, have revealed perfect transmission resonances in 1D magnonic quasicrystals^68^, thereby extending the understanding of deterministic aperiodicity in magnonic systems beyond the Fibonacci case.

**Fig. 1:**
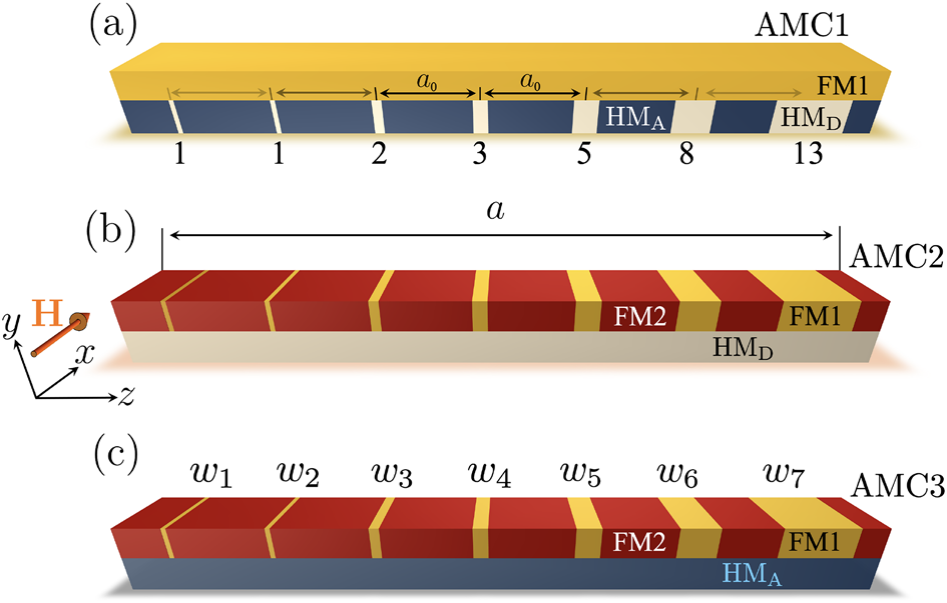
Schematic representation of 1D magnonic crystals based on the Fibonacci sequence within the unit supercell of size *a*. The positions of the stripe centers follow a period ($a_0$), while their widths ($w_n$) vary according to the Fibonacci rule. In panel (**a**), the system AMC1 consists of a ferromagnetic film (FM1) with a heavy metal HM$_\textrm{D}$, which induces both PMA and DMI. HM$_\textrm{D}$ stripes follow the Fibonacci sequence, while the remaining regions are covered with heavy metal HM$_\textrm{A}$, which induces only PMA. In panel (**b**), denoted as AMC2, two ferromagnetic layers (FM1 and FM2) are placed on top of an HM$_\textrm{D}$ layer. In panel (**c**), referred to as AMC3, ferromagnetic films are coupled with an HM$_\textrm{A}$ layer. In all cases, the spin waves propagate along *z*, so that $\textbf{k} = k \hat{z}$, with the external field ($\textbf{H}$) applied along *x* (Damon–Eshbach configuration).

This work focuses on Fibonacci-based aperiodic magnonic superlattices (AMCs) treated within a periodic approximant approach, in which a finite number of Fibonacci terms is encoded into an extended unit cell that is periodically repeated. For practical implementation, the structure is restricted to seven consecutive Fibonacci terms, which are sufficient to reproduce the essential aperiodic modulation within the supercell and capture the characteristic spectral features associated with Fibonacci ordering. The central novelty of this work lies in demonstrating how chirality, saturation magnetization contrast, and perpendicular magnetic anisotropy, combined with deterministic modulation implemented through the periodic approximant, cooperatively encode modal selectivity. This interplay leads to the emergence of flat bands confined to a well-defined frequency window, set by the minima of the spin-wave dispersions of the corresponding effective continuous films. Crucially, this frequency window can be tuned by an external magnetic field, providing a simple and efficient route to externally control and broaden the dispersionless-mode regime.

## Methods

The systems under consideration are shown in Fig. 1, where spin waves are assumed to propagate along the *z*-direction, while the in-plane equilibrium magnetization points along *x*, with *y* being the normal direction. The structures shown in Fig. 1a–c are composed of magnetic and nonmagnetic components in the form of stripes with a Fibonacci sequence within the unit supercell of size *a*. Therefore, the structures studied in this work should be regarded as periodic approximants of Fibonacci superlattices rather than true quasicrystals. This approach enables the investigation of localization and flat-band formation driven by deterministic aperiodic modulation within a framework compatible with the plane-wave method. The stripe centers are positioned with a fixed center-to-center spacing $a_0$, while the stripe widths $w_n$ vary according to seven terms of the Fibonacci sequence. Figure 1a depicts the first Fibonacci-based aperiodic magnonic crystal (AMC1), where a sequence of heavy-metal (HM$_\textrm{D}$) stripes, responsible for inducing interfacial DMI and perpendicular magnetic anisotropy (PMA), is patterned following the Fibonacci rule. The remaining surface is covered with a nonmagnetic material HM$_\textrm{A}$, which induces only PMA. Both nonmagnetic layers are coupled with a single ferromagnetic film (FM1), forming a hybrid system in which chirality and anisotropy are spatially modulated^44^. Figure 1b shows a second configuration, AMC2, where two ferromagnetic materials (FM1 and FM2) are sequentially patterned on top of a continuous HM$_\textrm{D}$ layer, preserving the quasiperiodic order within the unit supercell. This configuration enables the exploration of spin-wave behavior in a system with uniform DMI, but varying magnetic properties. The configuration AMC3 is presented in Fig. 1c, where a bicomponent system composed of FM1 and FM2 stripes follows the Fibonacci sequence and is deposited on a continuous HM$_\textrm{A}$ layer, producing spatial variations in PMA through the anisotropy field $\mu _0H_\mathrm{\perp }=2K_\mathrm{\perp }/M_\textrm{s}$, where $K_\mathrm{\perp }$ is the anisotropy constant and $M_\textrm{s}$ the saturation magnetization. Both DMI and PMA interactions, modulated through the Fibonacci pattern, play a central role in the spatial localization, spectral response, and modal differentiation of spin waves in the periodic approximants of Fibonacci superlattices.

Magnetization dynamics is studied using the Landau-Lifshitz (LL) equation of motion,

1 $$\begin{aligned} \dot{\textbf{M}} (\textbf{r},t) = -\mu _0\gamma \textbf{M}(\textbf{r},t)\times \textbf{H}^\textrm{eff}(\textbf{r},t), \end{aligned}$$

where the dot indicates time derivative, $\gamma$ is the absolute value of the gyromagnetic ratio, $\textbf{M}(\textbf{r},t)$ the magnetization vector, and $\textbf{H}^\textrm{eff}(\textbf{r},t)$ the effective field. Assuming small deviations around the equilibrium state, both the magnetization and the effective field can be separated into dynamic and static parts^81^. At the interfaces between the ferromagnet and the HM-wires, an interfacial Dzyaloshinskii-Moriya interaction arises^40–46^. Therefore, according to the plane-wave method, the DMI strength $D(\textbf{r})$ and the dynamic magnetization $\textbf{m}\left( \textbf{r},\,t\right)$ are expanded into Fourier series^82^, namely $D(\textbf{r})= \sum _{\textbf{G}} D_{\textbf{G}} e^{i \textbf{G} \cdot \textbf{r} }$ and $\textbf{m}\left( \textbf{r},\,t\right) = \sum _{\textbf{G}} \textbf{m}_{\textbf{G}} e^{i \left[ \left( \textbf{G} + \textbf{k} \right) \cdot \textbf{r} - \omega t \right] }$, the latter form following from Bloch’s theorem. Saturation magnetization, exchange length, and anisotropy are expanded in the same way. Here, $\omega =2\pi f$ is the angular frequency, and $\textbf{G}=G_z \hat{z}=(2\pi n /a) \hat{z}$ denotes the reciprocal lattice vector of a one-dimensional periodic structure, with *n* being an integer and *a* the periodicity of the unit supercell along the *z* axis (see Fig. 1). It should be noted that the unit supercell considered here is sufficiently large to include a quasiperiodic arrangement corresponding to seven terms of the Fibonacci sequence.

By following Refs.^41, 43^, the LL equation can be mapped into an eigenvalue problem, $i\,\frac{\omega }{\mu _{0}\,\gamma }\,\textbf{m}_{\textbf{G}}=\tilde{\textbf{T}}\, \textbf{m}_{\textbf{G}}$, where the dynamical matrix $\tilde{\textbf{T}}$ can be written as

2 $$\begin{aligned} \tilde{\textbf{T}}= \begin{pmatrix} \textbf{T}^{zz} & \textbf{T}^{zy}\\ \textbf{T}^{yz} & \textbf{T}^{yy} \end{pmatrix}. \end{aligned}$$

The elements $\textbf{T}^{\eta \eta '}$ depend on the effective fields associated with the different magnetic interactions. To develop a more realistic theoretical picture of the usual ferromagnetic/heavy-metal ultrathin layers^34,35^, the considered effective field is

3 $$\begin{aligned} \textbf{H}^\mathrm{{eff}}(\textbf{r},t)&= \textbf{H}_{0}(\textbf{r},t) + \textbf{H}_\textrm{s}(\textbf{r},t) + \textbf{H}_\mathrm{{ex}} + \textbf{H}_\mathrm{{dip}}(\textbf{r},t)+ \textbf{H}_\mathrm{{dm}}(\textbf{r},t), \end{aligned}$$

where $\textbf{H}_{0}$ is the applied external field, $\textbf{H}_\textrm{s}$ the perpendicular anisotropy field, $\textbf{H}_\mathrm{{ex}}$ the exchange field, $\textbf{H}_\mathrm{{dip}}$ the magnetostatic field and $\textbf{H}_\mathrm{{dm}}$ the Dzyaloshinskii-Moriya field. The matrix elements are listed in the References ^29,41,43,45^. Nevertheless, the Fourier coefficients require an additional consideration to account for the particular distribution of the Fibonacci sequence. This sequence can be addressed by the function $F_n$, which is defined as

4 $$\begin{aligned} F_0 = 0,\qquad F_1 = 1,\qquad F_n = F_{n-1} + F_{n-2}\quad (n \ge 2). \end{aligned}$$

Thus, the stripe widths are defined by $w_{n} =10 F_{n}$ nm with $n=1,...,7$, resulting in the specific values $w_{1}=10$ nm, $w_{2}=10$ nm, $w_{3}=20$ nm, $w_{4}=30$ nm, $w_{5}=50$ nm, $w_{6}=80$ nm, and $w_{7}=130$ nm. To implement these parameters, the quasiperiodic properties of the Fibonacci crystal are incorporated into the Fourier coefficients. For instance, the Fourier coefficient associated with the DMI is^30^

5 $$\begin{aligned} D_{\textbf{G}} = \frac{D}{a} \sum _{n=1}^{7} w_{n} \, \textrm{sinc} \left( \frac{w_{n}}{2} \textbf{G} \cdot \hat{z} \right) e^{-i z_{n} \textbf{G} \cdot \hat{z}}, \end{aligned}$$

where the stripe positions are fixed at regular intervals

6 $$\begin{aligned} z_n = a_0\,\left( n-\frac{1}{2}\right) \quad \text {for } n=1,...,7 \end{aligned}$$

with $a_0=200$ nm. These positions, combined with the Fibonacci-defined widths $w_n$ from Eq. (4), establish the common structural basis for the chiral magnonic examined in this study.

### Micromagnetic simulations

Micromagnetic simulations were performed using the GPU-accelerated code mumax$^{3}$^83,84^. The simulated geometry has dimensions $32.2\,\mathrm {\mu m}\times 64\,\textrm{nm}\times 1\,\textrm{nm}$ along the (*z*, *x*, *y*) axes, accommodating 23 unit supercells of length $a = 1400$ nm. The computational grid comprises $2^{15} \times 2^5 \times 1$ cells, resulting in a cell size of approximately $1 \times 2 \times 1\,\textrm{nm}^3$. Periodic boundary conditions are applied along the *z*- and *x*-directions to suppress spin-wave reflections and stabilize equilibrium magnetization along the *x*-axis. The system is initialized with uniform magnetization along *x* and subjected to a static magnetic field $\mu _0 H_0 = 250$ mT in the same direction. The Fibonacci sequence is implemented by modulating material parameters, such as the saturation magnetization $M_\textrm{s}$, exchange stiffness $A_\textrm{ex}$, uniaxial anisotropy constant $K_\mathrm{\perp }$, or Dzyaloshinskii–Moriya strength *D*, within periodically defined regions.

Spin waves are excited by a localized, space- and time-dependent magnetic field pulse

$$\textbf{b}_{\text {rf}} = \mu _0 h_0\,\text {sinc} \left[ 2\pi f_\textrm{c} (t - t_0)\right] \text {sinc}(k_0 z)\,\hat{y},$$

where $h_0 = H_0/10$ is the pulse amplitude, $f_\textrm{c} = 30$ GHz is the cut-off frequency, $t_0 = 49.99$ ps is the pulse center, and $k_0 = 2\pi /\lambda _0$ with $\lambda _0 = 40$ nm the spatial wavelength. Simulations are run for 40 ns, with magnetization snapshots recorded every 0.5 ps. A damping parameter $\alpha = 0.002$ is used. Dispersion relations are extracted via two-dimensional Fast Fourier Transforms (FFTs) in space and time, using the Python-based analysis packages oommfpy^85^ and ubermag^86^.

## Results

In the studied cases, the unit supercell has a size of $a = 1400$ nm. An external magnetic field of $\mu _0 H_0 = 250$ mT is applied along the *x*-axis, while spin waves propagate along the *z*-direction, consistent with the Damon–Eshbach geometry. The film thickness is fixed at $d=1$ nm, and the gyromagnetic ratio is $\gamma = 175.87$ GHz/T. The remaining material parameters, including the exchange stiffness, saturation magnetization, and interfacial anisotropy constants for the FM/HM$_\textrm{D}$ and FM/HM$_\textrm{A}$ stacks, are summarized in Table 1. Note that PMA and DMI arise from interfacial effects induced by the contact of ferromagnetic layers with metals like platinum (Pt), or ruthenium (Ru)^44,87,88^.

**Table 1:** Magnetic and structural parameters considered in theory and simulations.

Parameter	Value
Exchange constant material 1 ($A_{\text {ex}_1}$)	15.17 pJ/m
Exchange constant material 2 ($A_{\text {ex}_2}$)	26.96 pJ/m
Saturation magnetization material 1 ($M_{\text {s}_1}$)	900 kA/m
Saturation magnetization material 2 ($M_{\text {s}_2}$)	1200 kA/m
Perpendicular anisotropy constant FM/HM$_\textrm{D}$($K_{\perp }^{\text {D}}$)	328.5 kJ/m$^{3}$
Perpendicular anisotropy constant FM/HM$_\textrm{A}$($K_{\perp }^{\text {A}}$)	517.5 kJ/m$^{3}$
Interfacial Dzyaloshinskii-Moriya FM/HM$_\textrm{D}$(*D*)	1 mJ/m$^{2}$

**Fig. 2:**
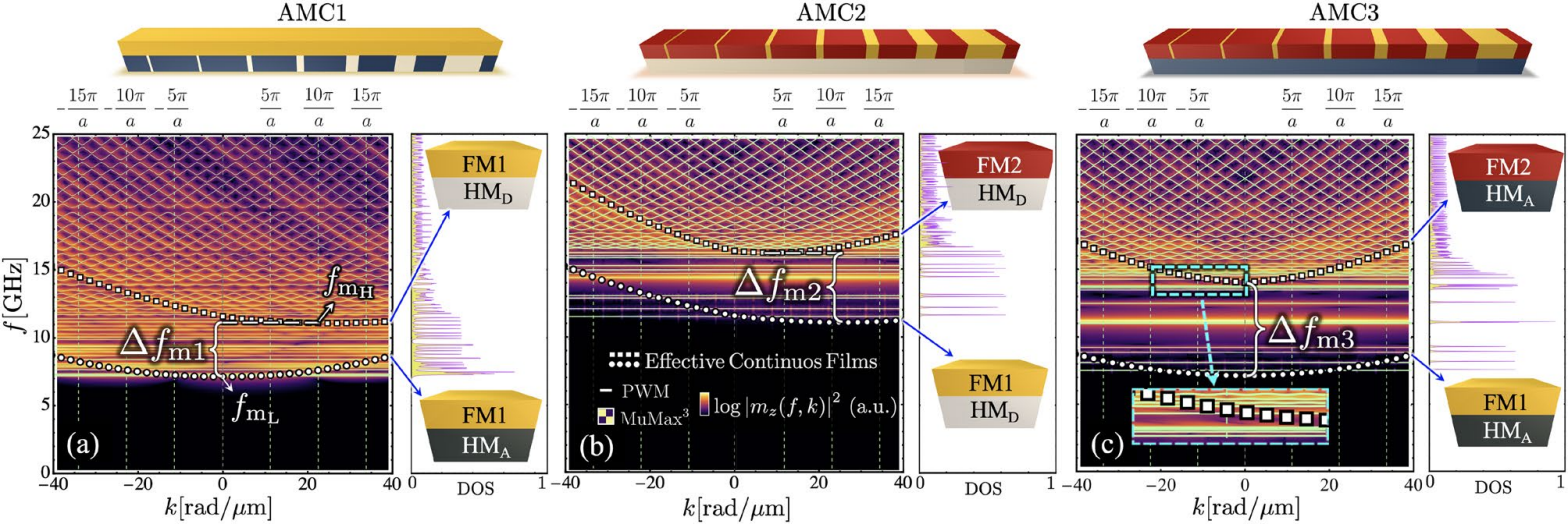
Spin-wave band structures and corresponding density of states (DOS) of chiral periodic approximants of Fibonacci superlattices with perpendicular magnetic anisotropy. Panel (**a**) shows results for the chiral magnonic crystal (AMC1), while panels (**b**,**c**) correspond to the bicomponent superlattices AMC2 and AMC3, respectively. Each band structure includes, for comparison, the spin-wave dispersions of the effective continuous films associated with the two constituent materials of the magnonic crystal (see circle and square markers). The quantities $f_\mathrm{m_L}$ and $f_\mathrm{m_H}$ denote the minimum frequencies of the effective-film dispersions with lower and higher frequency minima, respectively. The frequency window $\Delta f_\textrm{m}=f_\mathrm{m_H}-f_\mathrm{m_L}$ defines the spectral interval in which flat bands are excited and localized modes emerge. Results obtained using the plane-wave method (solid lines) are validated by full-scale micromagnetic simulations performed with MuMax$^3$ (color maps). Each column corresponds to a specific AMC configuration, as schematically illustrated above the panels.

The spin-wave spectra of periodic approximants of Fibonacci magnonic superlattices obtained in the absence of magnetic perpendicular anisotropy already exhibit several low-frequency flat bands, whose associated modes display varying degrees of spatial localization driven by material contrast and, in chiral configurations, by the presence of interfacial Dzyaloshinskii–Moriya interaction (see Sec. S1 in the Supplementary Material^89^). These results demonstrate that deterministic aperiodic modulation alone is sufficient to induce flat bands in magnonic superlattices. However, in the absence of anisotropy, the resulting localization remains relatively weak, and the flat bands are only marginally separated from dispersive bands, limiting both their spectral isolation and spatial confinement. For this reason, the analysis that follows focuses on configurations incorporating perpendicular magnetic anisotropy. The inclusion of anisotropy introduces an additional energy scale that cooperates with chirality and aperiodic material modulation to deepen the effective local frequency contrasts, thereby significantly enhancing flat-band formation and mode localization. This enhancement leads to well-defined frequency windows that host strongly confined modes and provides an experimentally accessible control parameter for engineering and tuning dispersionless magnon states. The anisotropic configurations, therefore, represent the most favorable and physically transparent regime for elucidating the localization mechanism and its potential for reconfigurable magnonic functionality.

In a more general description, particularly for ultrathin films of thickness $d=1$ nm, the effect of an interfacial perpendicular anisotropy constant $K_\mathrm{\perp }$ can be formally incorporated into an effective magnetization defined as $M_\textrm{eff}=M_\textrm{s}-2K_{\perp }/\mu _0M_\textrm{s}$. From this perspective, the flattening of the low-frequency modes may be interpreted as arising from the contrast in $M_\textrm{eff}$ between the constituent regions, rather than by anisotropy alone. In the present systems, the same interfacial anisotropy constant $K_\mathrm{\perp }$ acts on materials with different saturation magnetizations $M_\textrm{s}$, which enhances the resulting effective contrast. Nevertheless, for clarity and consistency of the physical discussion, in what follows, anisotropy will be referred explicitly as the control parameter, keeping in mind that, at this thickness scale, its effect is equivalently captured through the corresponding modification of the effective magnetization.

Figure 2a presents the SW spectrum calculated using the plane-wave method (lines) and micromagnetic simulations (color code), corresponding to the system depicted in Fig. 1a with $D = 1$ $\mathrm {mJ/m}^2$. Compared to the configuration without perpendicular anisotropy^89^, the introduction of PMA in the zones FM1/HM$_\textrm{D}$ (see Table 1) in the chiral AMC1 leads to a notable shift of the entire band structure toward lower frequencies. A clear excitation of low-frequency flat bands is observed, and the corresponding DOS displays well-defined, isolated peaks in this frequency range, confirming the presence of highly localized states. The physical origin of the frequency window within which the flat bands are predominantly excited can be understood by considering the spin-wave dispersions of the two effective continuous films associated with the constituent materials of the magnonic crystal. In the AMC1 system, these effective films correspond to the ferromagnetic bilayers FM1/HM$_\textrm{A}$ and FM1/HM$_\textrm{D}$. The corresponding dispersions are shown by circle and square markers in Fig. 2a, respectively. As observed, the frequency window $\Delta f_\textrm{m1}=f_\mathrm{m_H}-f_\mathrm{m_L}$, where $f_\mathrm{m_H}$ ($f_\mathrm{m_L}$) denotes the minimum of the higher- (lower-) frequency dispersion, coincides with the frequency range in which the flat bands are excited. Below the minimum of the lower-frequency dispersion ($f_\mathrm{m_L}$), spin-wave modes in the magnonic crystal are not supported. However, in the frequency interval lying between $f_\mathrm{m_L}$ and $f_\mathrm{m_H}$, dynamical modes can exist predominantly only in those regions whose local magnetic parameters are compatible with the lower-frequency dispersion of the effective film. In contrast, regions associated with the material exhibiting the higher-frequency dispersion (square markers in Fig. 2a) present a locally elevated resonance frequency that is incompatible with excitation within this interval defined by $\Delta f_\textrm{m1}$. This spectral mismatch leads to selective suppression of dynamics in high-frequency regions and, consequently, to strong spatial confinement of modes in low-frequency regions, resulting in dispersionless flat bands. In this sense, the frequency window $\Delta f_\textrm{m1}$ provides a key physical criterion for understanding and predicting the emergence of low-frequency flat modes and their associated localization patterns in Fibonacci magnonic superlattices. It is also shown that appropriate spatial modulation of saturation magnetization, anisotropy, and DMI leads to the formation of strongly localized spin-wave modes within the repeated unit supercell, with a characteristic localization length of approximately 100 nm. This strong real-space confinement, combined with the Bloch form of the eigenmodes, given by a periodic envelope multiplied by the phase factor $e^{ikz}$, with *k* restricted to the first Brillouin zone, provides an intuitive explanation for the emergence of flat bands. Since the smallest wavelength accessible within this zone is 2*a*, which is of the order of a few micrometers for the superlattice periods considered here, the Bloch phase varies negligibly over the spatial extent of the localized modes. As a result, the mode profile remains essentially unchanged across the Brillouin zone, and the corresponding eigenfrequency becomes nearly independent of the wave vector, giving rise to dispersionless flat bands.

**Fig. 3:**
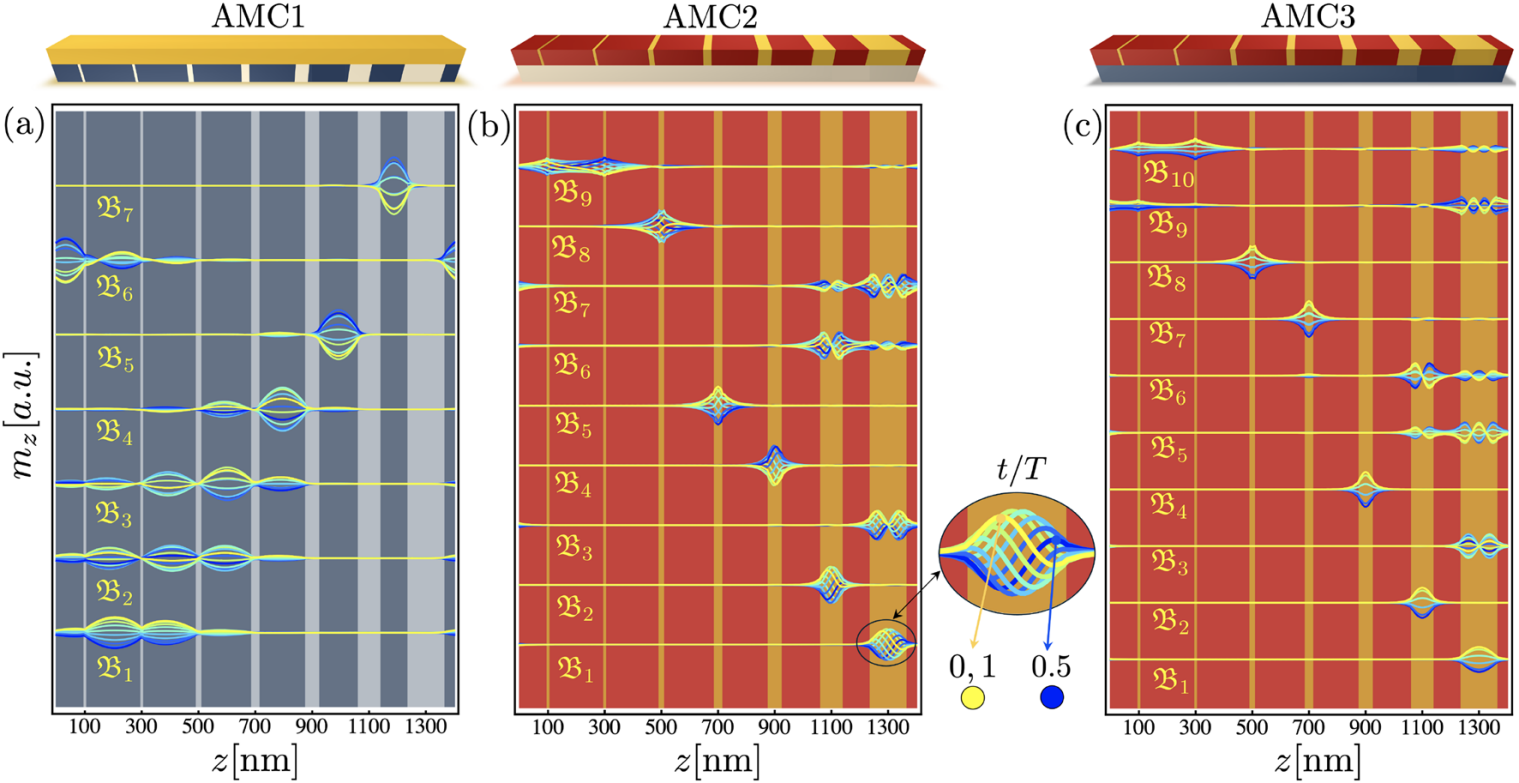
In-plane dynamic magnetization profiles ($m_z$) at $k = 0$ for the first spin-wave modes in (**a**) AMC1, (**b**) AMC2, and (**c**) AMC3, corresponding to the band structures shown in Fig. 2. Each panel displays the spatial envelope of the dynamic excitation within the Fibonacci unit supercell.

The band structure of the periodic approximant corresponding to the bicomponent system coupled to a continuous HM$_\textrm{D}$ film (AMC2) is analyzed in Fig. 2b, considering $K_{\perp }^{\text {D}} = 328.5~\text {kJ/m}^3$ and an interfacial DMI strength of $D = 1$ mJ/m$^2$. In this configuration, the regions with lower saturation magnetization (FM1) follow the Fibonacci sequence within the unit supercell. Similar to Fig. 2a, the inclusion of PMA results in the emergence of multiple flat modes in the lower-frequency bands. These highly localized states are evidenced in the corresponding density of states^44^. The frequency window $\Delta f_\textrm{m2}$ depicts the frequency range in which the flat bands are formed. Due to the interfacial DMI, the minima of the effective films are located at a finite wave vector for the low- and high-frequency dispersions^30,41,45^. Figure 2c shows bicomponent configurations in which perpendicular magnetic anisotropy is continuously introduced (bottom HM$_\textrm{A}$ layer). The corresponding DOS exhibits sharp and well-separated peaks, indicating the presence of highly localized states, similar to those observed in systems AMC1 and AMC2. Note that in Fig. 2c, the material FM1 (which has lower saturation magnetization than material FM2) follows the Fibonacci sequence; nonetheless, if the magnetic materials are reversed, the number of flat bands is substantially increased (see supplementary material). This behavior can be understood in terms of an exchange-dominated quantization rule. When the magnetic materials are reversed, the regions characterized by reduced saturation magnetization, which define the local low-frequency wells, occupy wider stripe segments within the supercell. Since the flat-band modes are confined in these low-frequency regions, the increased stripe width allows the accommodation of a larger number of standing-wave-like excitations across the stripe width. In this regime, the spatial quantization is governed predominantly by the exchange interaction (and modified by DMI in chiral configurations). Consequently, wider low-$M_\textrm{s}$ regions support additional quantized states, leading to a significant increase in the number of flat bands within the same frequency window. The frequency range defined by $\Delta f_\textrm{m3}$ explains the reason for the flat-band formation and the localization in the $FM1/HM_A$ zones.  

 It should be noted that, although the frequency window $\Delta f_\textrm{m}$ identifies the primary regime where flat bands with strong spatial localization systematically emerge, weakly dispersive or nearly flat modes may also appear outside this interval, as shown in the inset of Fig. 2c. However, these modes generally exhibit a lower degree of localization than the lower-frequency ones. It is worth noting that the lowest-frequency flat mode in Fig. 2a–c appears at a frequency slightly above $f_\mathrm{m_L}=f(k^*)$, which is defined for the corresponding effective continuous film and where $k^*$ corresponds to the wave vector at the frequency minimum. This behavior arises because the widest stripe, $w_7$, where the spin waves of the lowest-frequency branch are localized, has a finite width that is smaller than the characteristic wavelength associated with the dispersion minimum. In the case shown in Fig. 2b, the characteristic wavelength is $\lambda ^* = 2\pi /k^*=273$ nm, while $w_7 = 130$ nm. As a result, the lowest-energy mode cannot fully accommodate its wavelength within this region, leading to an upward frequency shift relative to $f_\mathrm{m_L}$. Upon increasing the stripe width $w_7$, the lowest-frequency mode systematically shifts toward $f_\mathrm{m_L}$ (see Fig. S2.1 in the Supplementary Material^89^). In the configuration where $f_\mathrm{m_L}$ occurs at $k=0$ (i.e., in the absence of DMI), the lowest-frequency flat mode also lies above $f_\mathrm{m_L}$ (see circles in Fig. 2a,c). This behavior is expected, since the corresponding excitation on the widest stripe approaches a nearly uniform mode with $k \approx 0$ (and $\lambda \rightarrow \infty$). However, due to the modulation of the dynamic magnetization at the stripe edges, the mode is not perfectly uniform and is then excited at a slightly higher frequency.     

The incorporation of out-of-plane anisotropy in Fibonacci-based configurations significantly enhances the formation of flat bands at low frequencies, indicating a stronger spatial localization of the corresponding spin-wave modes. To examine this enhanced localization, the spatial distribution of the dynamical magnetization is analyzed. Special attention is paid to the in-plane magnetization component $m_{z}$ at $k=0$, which provides a direct real-space visualization of the spin-wave mode profiles. These profiles reveal how the combined effects of geometric modulation and the distribution of anisotropy shape the spin-wave envelopes and determine their degree of spatial confinement. As illustrated in Fig. 3a, the lowest seven modes of system AMC1 exhibit pronounced spatial localization within the supercell. The spin-wave amplitudes are predominantly confined to the darker FM1/HM$_\textrm{A}$ regions, indicating that these zones act as preferential localization sites for the excitations. In particular, the lowest-frequency mode $\mathfrak {B}_1$ is strongly concentrated beneath the wider FM1/HM$\textrm{A}$ stripes. Importantly, this spatial confinement persists even for higher-order modes, for which the oscillation amplitude remains largely concentrated within the FM1/HM$\textrm{A}$ regions. This behavior reflects the local reduction of the effective spin-wave frequency induced by the enhanced PMA associated with HM$\textrm{A}$. Consequently, these regions act as effective potential wells for magnons, in agreement with the continuous-film behavior shown in Fig. 2a, where the HM$_\textrm{A}$-induced anisotropy leads to a lower local resonance frequency. For the AMC3 structure (Fig. 3c), the flat-mode profiles likewise exhibit localization within the low-$M_\mathrm{{s}}$ regions. This behavior can again be analyzed in terms of the frequency window $\Delta f_\textrm{m3}$, which is defined by the minima of the dispersions of the corresponding effective continuous films. Within this window, spin-wave excitations are energetically allowed only in regions with compatible local magnetic parameters, leading to selective spatial confinement. If the materials are reversed in structures AMC2 and AMC3 ($\textrm{FM1}\rightarrow \textrm{FM2}$ and $\textrm{FM2}\rightarrow \textrm{FM1}$), an even larger number of flat bands emerges. This behavior arises because the material with reduced saturation magnetization occupies a larger fraction of the structure, thereby allowing the excitation of a greater number of low-frequency, well-localized flat modes, as shown in ^89^.

It is worth noting that the spin-wave wavelengths extracted from the corresponding continuous film at the flat-band frequencies do not correlate with the widths of the stripe regions where the modes localize. This observation indicates that the localization cannot be described by a simple standing-wave condition set solely by the stripe width. Rather, the confinement is strongly influenced by the effective boundary conditions at interfaces between regions with different magnetic properties, including spatial anisotropy contrast, interfacial DMI, and resulting material-dependent magnetic discontinuities. Overall, these results demonstrate that reduced local spin-wave frequencies, arising from anisotropy and saturation magnetization contrasts, play a central role in driving flat-band formation and mode localization in Fibonacci superlattices.

**Fig. 4:**
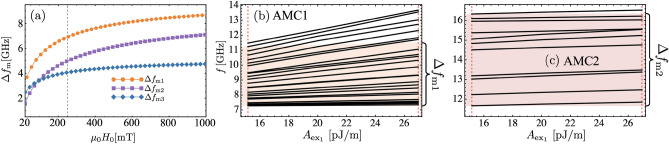
(**a**) Frequency window $\Delta f_\textrm{m}$ as a function of the external magnetic field for the different AMC configurations, illustrating the tunability of the flat-band regime. The vertical dashed line indicates the field value $\mu _0 H_0 = 250$ mT used in Fig. 2. (**b**,**c**) Evolution of the flat-band modes within the frequency windows $\Delta f_\textrm{m1}$ and $\Delta f_\textrm{m2}$ as a function of the exchange stiffness $A_\mathrm{ex_1}$.

From the previous results, it is evident that $\Delta f_\textrm{m}$ governs the emergence of flat bands at low frequencies. Importantly, this frequency window is determined by the spin-wave dispersions of the corresponding continuous films, making it straightforward to analyze and tune in response to external perturbations, such as an applied magnetic field. The dependence of $\Delta f_\textrm{m}$ on the external field is shown in Fig. 4a. Consequently, the flat-band formation can be externally controlled and enhanced by applying a magnetic field, thereby broadening the frequency range over which dispersionless modes are excited. It is important to note that, despite the presence of PMA, the equilibrium magnetization remains saturated along the in-plane *x* direction for the field range considered in Fig. 4a. This is because the demagnetizing (shape) energy of the ultrathin film overcomes the perpendicular anisotropy contribution in most of the cases ($\mu _0 M_\textrm{s} > 2K_{\perp }/M_\textrm{s}$), except in the case FM1/HM$_\textrm{D}$ where $\mu _0 M_\textrm{s} \approx 2K_{\perp }/M_\textrm{s}$. As a result, the effective anisotropy favors an in-plane magnetization configuration, which remains stable even at a small external magnetic field. In Fig. 4b,c, the evolution of the flat modes within the frequency windows $\Delta f_\textrm{m1}$ and $\Delta f_\textrm{m2}$ ( at $k=0$) is analyzed as a function of the exchange constant $A_\mathrm{ex_1}$. The lowest-frequency flat modes remain largely insensitive to variations of the exchange constant. An exception is found in the AMC1 configuration, where the flat modes located close to the upper bound of the frequency window, i.e., near the frequency minimum $f_\mathrm{m_H}$, exhibit a noticeable upward frequency shift as $A_\mathrm{ex_1}$ increases. This behavior is attributed to the nature of the corresponding excitations. Higher-order flat modes have more internal nodes within the FM1/HM$_\textrm{A}$ regions, leading to enhanced spatial gradients in the dynamic magnetization. As a consequence, these modes are more strongly affected by the exchange interaction, which increases under spatial variations and shifts their frequencies upward with increasing $A_\mathrm{ex_1}$. In contrast, the lowest flat modes exhibit smoother spatial profiles and fewer nodes, rendering them significantly less sensitive to exchange-driven energy contributions. Also, in Fig. 4b, it is noted that some modes lie outside the $\Delta f_\textrm{m1}$ range as the exchange interaction increases. In this case, the modes are no longer localized excitations but rather dispersive bands (not shown).

## Discussions

The previous results show that the Fibonacci-based magnonic crystals naturally encode modal selectivity by shaping the spatial distribution of spin-wave excitations. Their intrinsic geometric order generates modes whose profiles are neither fully extended nor strictly localized, but instead reflect complex localization governed by the underlying structure. In the previous calculations, the anisotropy constants ($K_\mathrm{\perp }^\textrm{A,D}$) were sufficiently large to ensure strong perpendicular anisotropy, leading to the confinement of localized modes beneath the nonmagnetic stripes. In contrast, when the anisotropic contributions are weak, such localization effects do not occur, and consequently, the formation of flat bands at low frequencies is not expected^45^.

Although the spatial localization of spin-wave modes can be understood in terms of effective potential wells arising from local reductions of the spin-wave frequency, the deterministic order introduced by the Fibonacci sequence plays a distinct and essential role. Rather than merely confining modes, the Fibonacci-based modulation organizes a hierarchy of non-equivalent local environments within the unit supercell, thereby encoding where and which modes localize. In contrast to periodic structures composed of identical confinement regions, this arrangement yields reproducible yet non-periodic localization patterns and a dense set of flat or weakly dispersive bands with distinct spatial profiles. In chiral configurations, the presence of interfacial Dzyaloshinskii–Moriya interaction further enriches this behavior by introducing nonreciprocity and a time-dependent redistribution of the standing waves, as shown in Fig. 3b.

Overall, the theoretical predictions presented herein are consistent with the experimental observations of tunable flat bands and nonreciprocity in chiral Fibonacci magnonic crystals^72^. Nonetheless, in the present work, the plane-wave method provides a tractable and physically transparent framework for unveiling the fundamental mechanisms underlying flat-band formation and spatial localization. Through this approach, it becomes possible to disentangle the individual contributions of interfacial DMI, perpendicular magnetic anisotropy, and saturation magnetization contrast, thereby establishing general design principles that extend beyond purely DMI-driven phenomena. In particular, the analysis demonstrates that the emergence and tunability of flat modes can be systematically understood in terms of the frequency window $\Delta f_\textrm{m}$, defined by the minima of the effective-film dispersions. This concept extends the design space toward anisotropy- and $M_{\textrm{s}}$-driven regimes, enabling predictive control over the spectral position and spatial confinement of localized spin-wave modes in magnonic systems based on Fibonacci superlattices.

It is worth mentioning that the physical mechanism underlying the emergence of low-frequency flat bands and the associated mode localization does not rely on any special arithmetic property of the Fibonacci sequence within the unit supercell. Instead, it is governed by the spatially inhomogeneous landscape of geometric and magnetic parameters—such as *D*, perpendicular anisotropy, and $M_{\textrm{s}}$—which defines local frequency minima and a tunable flat-band window $\Delta f_\textrm{m}$ set by the minima of the corresponding effective-film dispersions. Consequently, replacing the Fibonacci order with another aperiodic arrangement yields qualitatively similar phenomenology: an enhanced density of nearly dispersionless bands, and selective confinement of the lowest modes to high-contrast regions (DMI-active or low-$M_{\textrm{s}}$/high-PMA zones). Thus, the Fibonacci pattern is primarily adopted for clarity and reproducibility. It is a well-studied aperiodic order that facilitates systematic finite-cell constructions and straightforward comparison with previous magnonic literature. In the present implementation, a finite inflation of the Fibonacci sequence (seven terms) is sufficient to capture the essential aperiodic modulation while keeping the plane-wave basis tractable.

In conclusion, Fibonacci-based magnonic superlattices offer a deterministic route to spatial-mode control and flat-band engineering at low frequencies. Across chiral and non-chiral architectures (AMC1–AMC3), plane-wave theory corroborated by full micromagnetic simulations reveals that modal localization is governed by material contrasts, perpendicular anisotropy, and chiral Dzyaloshinskii–Moriya interaction, concentrating amplitudes either in DMI-active regions or in low-$M_{\mathrm s}$ zones, and that perpendicular anisotropy markedly enhances the density and confinement of flat modes. Crucially, the frequency window that allows flat-band formation, $\Delta f_\textrm{m}$, increases with the applied field, providing direct external control to expand and strengthen the regime in which dispersionless modes are excited. These results position Fibonacci-based aperiodic magnonic crystals as compact, reconfigurable building blocks for filters, multiplexers, and logic magnonic elements.

## Supplementary Information

Supplementary Information.

## Data Availability

The data that support the findings of this study are available from the corresponding author upon reasonable request.
