# Supplementary material for: Mode localization in chiral periodic approximants of Fibonacci magnonic superlattices
Source: Sci Rep. 2026 Mar 27;16:10924. doi: 10.1038/s41598-026-44837-2 (PMC13039711; doi:10.1038/s41598-026-44837-2)
Supplement: Supplementary file 1 — Supplementary Information. [file 41598_2026_44837_MOESM1_ESM.zip › Supplementary_Material_Fibonnacci.pdf]

Supplementary material: Mode Localization in Chiral Periodic Approximants of Fibonacci Magnonic Superlattices

J. Flores-Farías[1], P. Contreras-Gallardo[2], F. Brevis[2], N. Vidal-Silva[3], P. Landeros[2], R. Arias[1,4], and R. A. Gallardo[2,*]

[1]Departamento de Física, Facultad de Ciencias Físicas y Matemáticas, Universidad de Chile, Santiago, Chile
[2]Departamento de Física, Universidad Técnica Federico Santa María, Avenida España 1680, Valparaíso, Chile
[3]Departamento de Ciencias Físicas, Universidad de La Frontera, Casilla 54-D, Temuco, Chile

[4]Center for the Development of Nanoscience and Nanotechnology (CEDENNA), 9170022 Santiago, Chile

[*]rodolfo.gallardo@usm.cl

This Supplementary Material provides additional spin-wave band structures, density-of-states analyses, and mode profiles supporting the main results on mode localization in periodic approximants of Fibonacci superlattices. The analysis also shows how the lowest flat-band frequency depends on the width of the widest stripe.

S1 Band structure and profiles for Fibonacci superlattices without perpendicular anisotropy

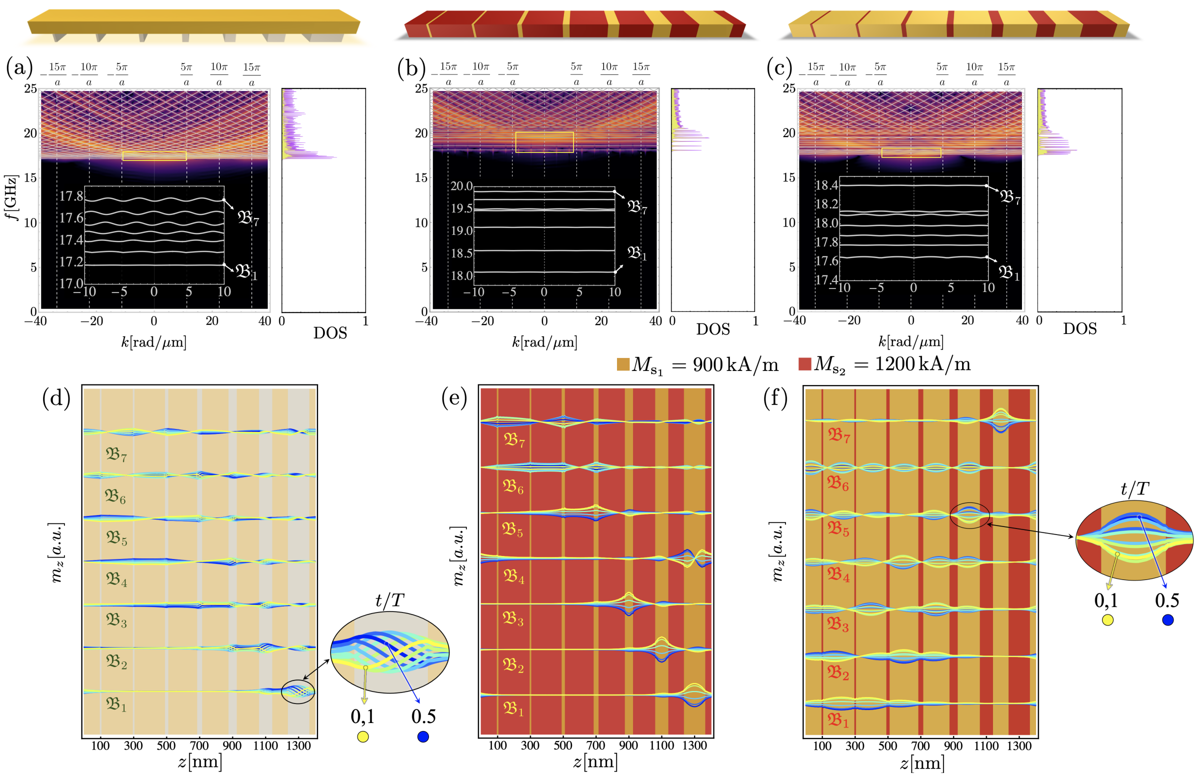

Figure S1.1. Magnonic band structures and corresponding density of states (DOS) for several configurations of Fibonacci superlattices with zero PMA, computed under a uniform magnetic field of $\mu_0 H_0 = 250$ mT applied along the x direction. (a) AMC1 incorporating interfacial Dzyaloshinskii–Moriya interaction with $D = 1$ mJ/m^2 and a uniform saturation magnetization of $M_{s_1} = 900$ kA/m. (b–c) Bicomponent MCs composed of two ferromagnetic materials with M_{s_1} and $M_{s_2} = 1200$ kA/m. Insets in panels (a–c) show an enlarged view of the low-frequency region, highlighting isolated modes and flat bands among the first spin-wave branches ($\mathfrak{B}_n$). Panels (d–f) present the corresponding spatial profiles of the in-plane dynamic magnetization component (m_z) at $k = 0$, illustrating the mode localization patterns within the extended unit cell. The time evolution of spin waves is analyzed using their envelope, which is calculated as a function of the dimensionless time t/T, where T denotes the characteristic oscillation period.

Fig. S1.1 presents the spin-wave band structures and the in-plane dynamic magnetization profiles for both chiral and bicomponent Fibonacci superlattices. The analysis first focuses on configurations without out-of-plane anisotropy, enabling a direct evaluation of the effects arising exclusively from the quasiperiodic magnetic components and the interfacial DMI. In Fig. S1.1(a), a chiral magnonic crystal (MC) with interfacial DMI strength $D = 1$ mJ/m^2 and $M_{s_1} = 900$ kA/m is shown. The spin-wave spectra exhibit a series of low-frequency bands confined within a narrow frequency window. The inset shows a magnified view highlighting well-separated modes, with the lowest band exhibiting dispersionless behavior, indicative of strong spatial localization. This spectral structure is further supported by the calculation of the corresponding density of states (DOS), which reveals a high concentration of states in this frequency interval, confirming the presence of multiple confined modes.

For the bicomponent Fibonacci quasicrystals, two distinct configurations are analyzed, selecting $M_{s_1} = 900$ kA/m and $M_{s_2} = 1200$ kA/m, where in Fig. S1.1(b) [Fig. S1.1(c)] the material FM1 [material FM2] follows the Fibonacci sequence. In Fig. S1.1(b), several low-frequency bands appear as flat modes, exhibiting strong spatial localization, as confirmed by the corresponding peaks in the DOS. In Fig. S1.1(c), where material 2 is arranged according to the Fibonacci sequence, the MC exhibits a larger number of flat modes in the low-frequency range, distributed over a narrower spectral interval. The associated DOS reveals that the individual peaks are no longer clearly resolved, indicating a denser clustering of flat modes.

To provide a more detailed insight into the spin-wave behavior in these quasicrystals, the SW mode profiles are analyzed. Figs. S1.1(d–f) show the dynamic magnetization profiles (represented by the in-plane dynamic magnetization component m_z) evaluated at $k = 0$. The seven lowest-frequency bands ($\mathfrak{B}_n$, with $n = 1$ to $n = 7$) are considered. In all cases, the dynamic magnetization is evaluated at different times to visualize the corresponding wave envelopes. In Fig. S1.1(d), only the lowest-frequency mode exhibits pronounced localization in the region where the two wider heavy-metal (HM) stripes are present. The higher-frequency bands do not show significant spatial localization, as their envelopes are distributed throughout the extended unit cell. The localization behavior of the lowest-frequency mode is consistent with previous observations in chiral magnonic crystals interfaced with periodic arrays of HM stripes, where low-frequency modes localize at FM/HM interfaces. Additionally, the nontrivial temporal evolution of the modes is evidenced by the color map, which reveals a time-dependent shift of the magnetization maxima within the ferromagnetic regions adjacent to the HMs. This behavior reflects a dynamical redistribution of the magnetization amplitude and phase, arising from the underlying chiral coupling at the interfaces.

Figs. S1.1(e) and S1.1(f) present the magnetization profiles for the two bicomponent Fibonacci configurations. In Fig. S1.1(e), the regions following the Fibonacci sequence are assigned the lower saturation magnetization. In this case, the lowest-frequency mode exhibits strong localization within the first wider stripe ($w_7 = 130$ nm), while the second and third low-frequency modes are predominantly localized in the second (around $z = 400$ nm) and third (around $z = 200$ nm) wider stripes, respectively. As the frequency increases (for bands $\mathfrak{B}_n$ with $n > 3$), the SW modes become progressively less confined, and the excitation envelope spreads across the entire unit cell. Fig. S1.1(f) corresponds to the inverse configuration, where the higher saturation magnetization is assigned to the Fibonacci stripes. Here, the spin-wave modes initially display a more spatially distributed localization pattern, with enhanced magnetization amplitude in the regions of lower saturation magnetization. As the frequency increases, the SW envelope gradually becomes more localized, concentrating on specific regions of the structure. This behavior is particularly evident for the band $\mathfrak{B}_7$ in Fig. S1.1(f), which exhibits a dispersionless character due to strong spatial confinement. It is worth noting that, unlike the SW profiles of the chiral MC shown in Fig. S1.1(d), the temporal evolution of the modes (color code) in the bicomponent structures follows conventional standing-wave oscillations, with maxima located at fixed spatial positions. This difference is a direct consequence of the nonchiral nature of the bicomponent configuration.

S2 Evolution of the lowest flat-band mode with the stripe width

The evolution of the lowest-frequency flat mode as a function of the widest stripe width w_7 is presented in Fig. S2.1(a), where the frequency position of this mode is extracted for different values of w_7. A clear monotonic trend is observed: as the stripe width increases, the frequency of the lowest flat mode decreases systematically. This behavior can be understood in terms of a finite-size confinement effect associated with the characteristic wavelength $\lambda^* = 2\pi/k^* \approx 273$ nm, where k^* denotes the wave vector at which the dispersion of the effective continuous film reaches its minimum frequency $f_{m_L} = f(k^*)$. As schematically illustrated in Fig. S2.1(b), when the stripe width w_7 is smaller than λ^*, the spatial extent of the region supporting the localized mode is insufficient to fully accommodate a spin-wave excitation with wavelength λ^*. Consequently, the lowest-energy mode is forced into a more confined spatial profile, leading to an upward shift of its resonance frequency relative to f_{m_L}. For example, in the narrow-stripe case $w_7 = 80$ nm, the lowest flat mode is excited at a frequency approximately 0.9 GHz above f_{m_L}. As w_7 increases, the confinement constraint is progressively relaxed, allowing the localized mode to more closely reproduce the spatial profile associated with the dispersion minimum. As a result, the frequency of the lowest flat mode continuously shifts downward and asymptotically approaches f_{m_L}. This analysis demonstrates that the lowest-frequency flat mode is not solely governed by the spectral-mismatch mechanism discussed in the main text, but is also quantitatively controlled by the interplay between the characteristic wavelength of the effective continuous film and the finite spatial extent of the stripe where localization occurs. In the limit $w_7 \gg \lambda^*$, the finite-size effect becomes negligible, and the flat mode converges to the minimum

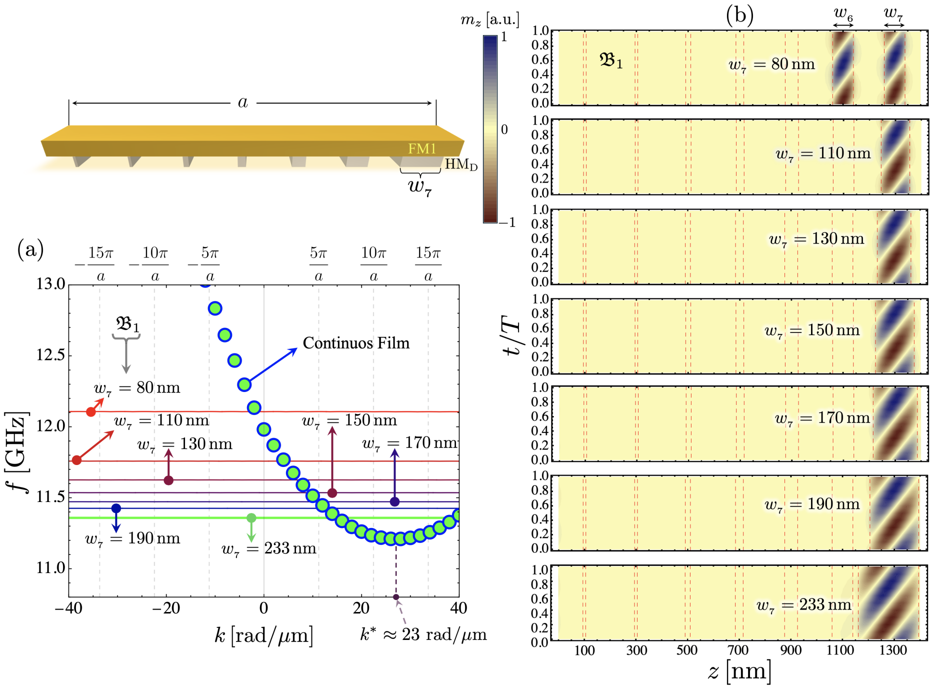

Figure S2.1. Evolution of the lowest-frequency flat-band mode as a function of the widest stripe width w_7. (a) Frequency of the lowest flat mode extracted from the spin-wave band structure for different values of w_7. As the stripe width increases, the mode frequency monotonically decreases and approaches the minimum frequency $f_{m_L} = f(k = k^*)$ of the corresponding effective continuous film. (b) Spin-wave profiles represented by the normal dynamic component m_z. For narrow stripes ($w_7 \ll \lambda^*$), the characteristic wavelength $\lambda^* = 2\pi/k^*$ associated with the dispersion minimum cannot be fully accommodated within the stripe, resulting in an upward frequency shift of the localized mode. As w_7 increases, the spatial confinement constraint is progressively relaxed, allowing the mode to better match the dispersion minimum and leading to convergence toward f_{m_L}.

frequency of the corresponding continuous-film dispersion.

S3 Band structure and profiles for Fibonacci superlattices with perpendicular anisotropy

The analysis now focuses on the periodic approximants of Fibonacci superlattices with perpendicular magnetic anisotropy (PMA), namely AMC2 and AMC3, where the ferromagnetic material FM2 follows the Fibonacci sequence. The first configuration is shown in Fig. S3.1(a), corresponding to a bicomponent magnonic crystal coupled to an HM$_D$ layer. In this case, a clear increase in the number of flat bands is observed compared with the configuration in which FM1 follows the Fibonacci sequence. The markers in Fig. S3.1(a) indicate the frequency interval Δf_{m2}, defined by the separation between the minima of the dispersions of the two effective continuous films. This interval hosts the localized flat modes, evidencing that the same spectral-mismatch mechanism discussed in the main text remains operative in the presence of perpendicular anisotropy.

In Fig. S3.1(b), where M_{s_2} occupies the Fibonacci stripes, the bicomponent configuration includes the anisotropy induced by the heavy-metal layer HM$_A$, which generates PMA but does not induce interfacial DMI. Compared to the bicomponent superlattice in which FM1 follows the Fibonacci sequence, an even larger number of flat bands emerges in the low-frequency region. In both cases shown in Fig. S3.1(a) and Fig. S3.1(b), the increased number of flat modes can be understood from the larger fraction of FM1 material present in the unit cell, which provides a greater spatial extent capable of supporting multiple low-frequency localized modes.

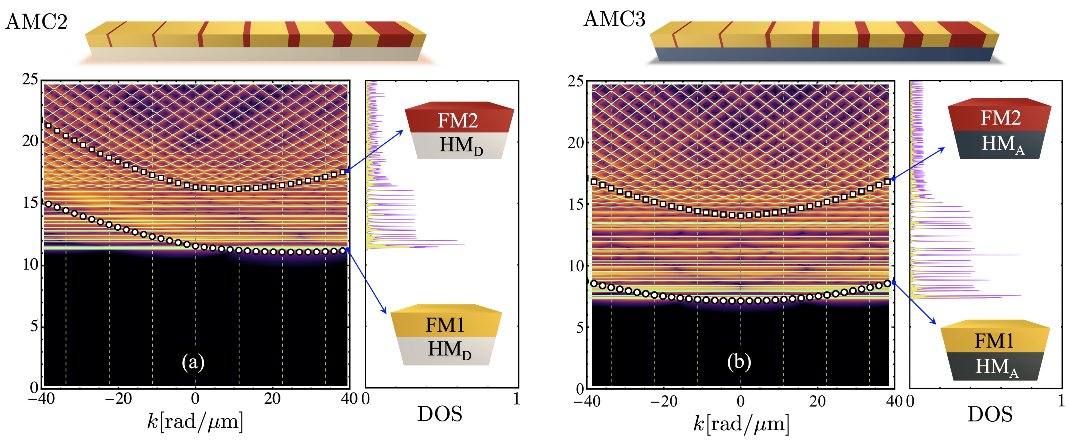

Figure S3.1. Spin-wave band structures and corresponding density of states (DOS) for different configurations of Fibonacci magnonic superlattices incorporating perpendicular magnetic anisotropy. Panels (a) show results for the chiral quasicrystal (AMC2, with $\mathrm{HM_D}$), while panels (b) correspond to the bicomponent quasicrystals (AMC3, with $\mathrm{HM_A}$). In all cases, the dispersion of the corresponding effective continuous film is included as a reference. The results were obtained using the plane-wave method (solid lines) and micromagnetic simulations (color maps).
