# Supplementary figures and images for: Mode localization in chiral periodic approximants of Fibonacci magnonic superlattices
Source: Sci Rep. 2026 Mar 27;16:10924. doi: 10.1038/s41598-026-44837-2 (PMC13039711; doi:10.1038/s41598-026-44837-2)

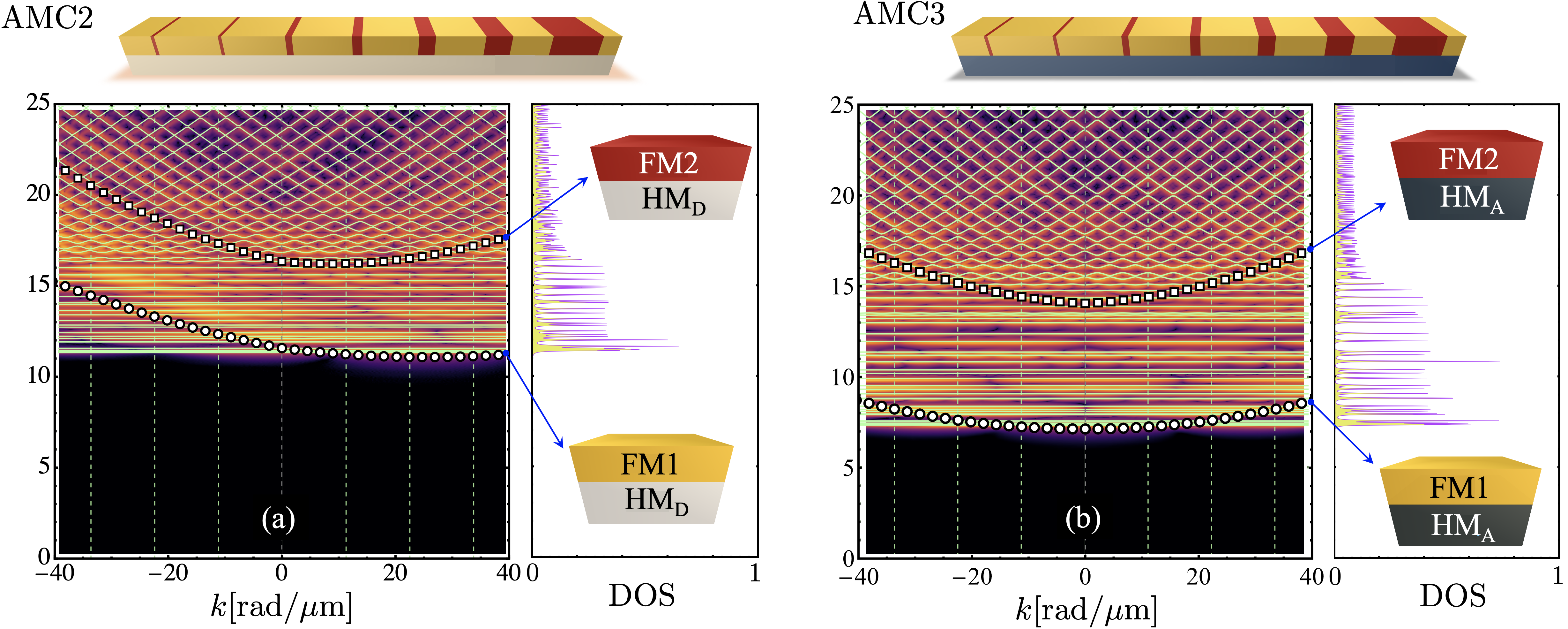

Supplement: Supplementary file 1 — Supplementary Information. [file 41598_2026_44837_MOESM1_ESM.zip › FigS3.png]

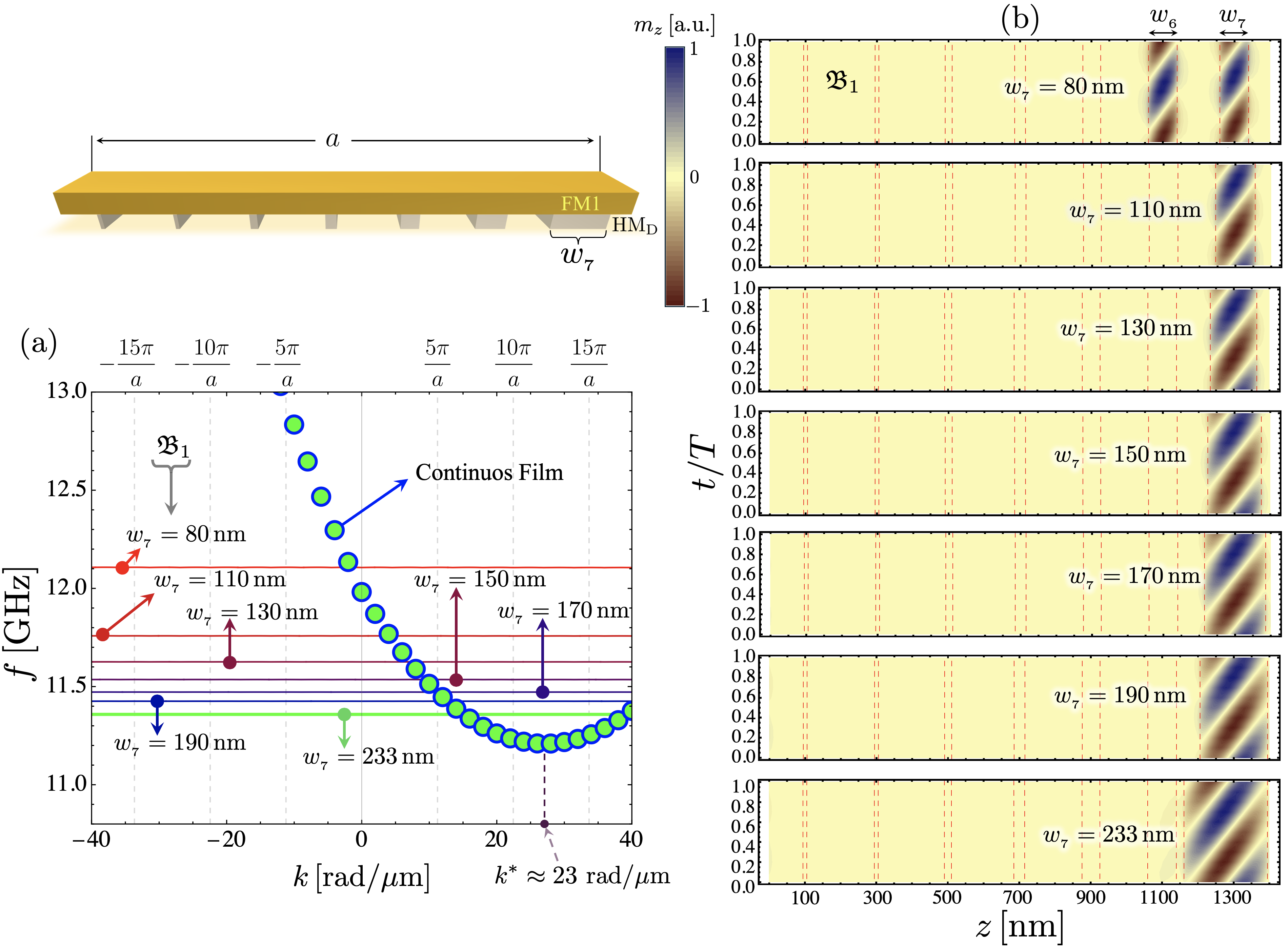

Supplement: Supplementary file 1 — Supplementary Information. [file 41598_2026_44837_MOESM1_ESM.zip › FigS2.png]

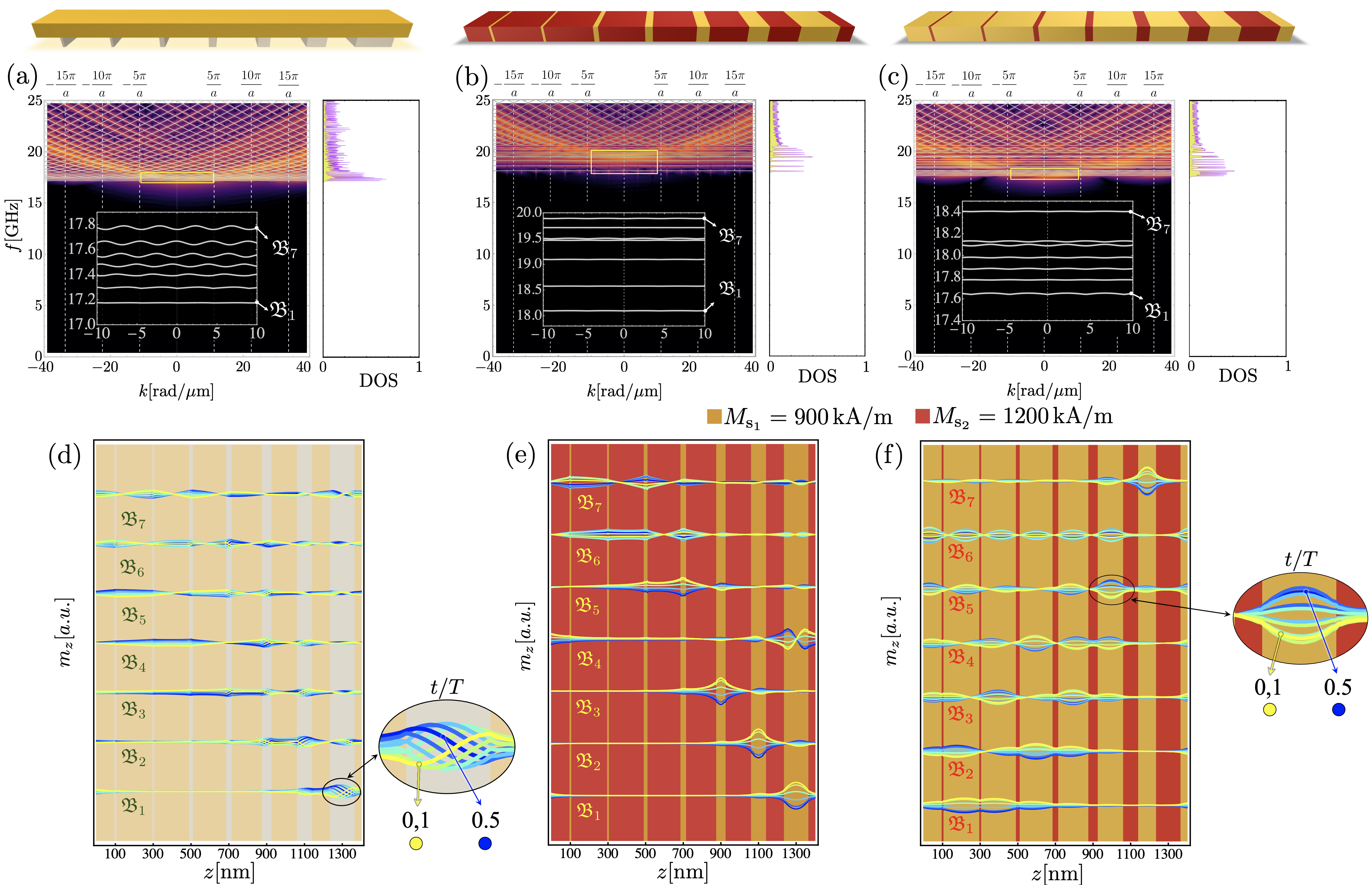

Supplement: Supplementary file 1 — Supplementary Information. [file 41598_2026_44837_MOESM1_ESM.zip › FigS1.png]
